# Fourth Annual Meeting at Manchester

**Published:** 1919-06-21

**Authors:** 


### THE COLLEGE OF NURSING.

### FOURTH ANNUAL MEETING AT MANCHESTER.

### Sir Arthur Stanley's Speech.

[We are able to give the following complete abstract of the Chairman's chief points.—Ed. The Hospital.]

The fourth annual report of the College of Nursing, which you have all received, begins by expressing satisfaction with the results achieved by the work of the year under review, and I venture to think that you will agree that this satisfaction is warranted.

The progress of the College of Nursing, Ltd., has indeed been remarkable, and is a clear proof of the urgent need for an association such as this, which should be truly representative of nurses and be able to voice their opinions.

It will be within your memory that the College only came into being at the end of 1915. By March 31, 1917, we had 2,553 members; by March 31, 1918, we had nearly 8,000 members; by March 31, 1919, we had 13,047 members. This shows a very satisfactory state of things, and if all the nurses who are at present members of the College will try to get new members during the year we shall very soon reach the figure of 20,000, at which we are aiming.

I am glad to say that the finances of the College are even more satisfactory. By March 31, 1917, we had £2,800 8s. 9d.; by March 31, 1918, we had £8,032 6s. 1d.; and by March 31, 1919, we had no less than £40,421 8s. 2d. This satisfactory result is largely due to the assistance that we have received from the British Women's Hospital and from Miss May Beeman. I am sure that you would wish me in your name to express our very deep and sincere gratitude to these ladies who have thus come to our assistance, especially in view of the fact that they have been subjected to violent abuse for so doing. To Dame May Whitty (the Chairman), Lady Cowdray (the Honorary Treasurer), and Miss Lilian Braithwaite (the Honorary Secretary), of the Nation's Fund for Nurses, we would tender our hearty thanks.

In this connection I should like to say a few words about the Nation's Fund for Nurses. You are all of you aware that this Fund was started for two purposes—firstly, to provide an endowment fund for the College of Nursing; and secondly, to make adequate provision for those nurses who have fallen upon evil days. With regard to the College Endowment Fund, I am happy to tell you that we have got about half the amount at which we are aiming, and I hope that we shall be able to get the remaining half during the next twelve months. When that is done the intention is that the Nation's Fund for Nurses shall cease its efforts on behalf of the College of Nursing, and the Fund shall be devoted entirely to the assistance of individual nurses who stand in need of help, and to the promotion and furtherance of schemes for the benefit of nurses generally. The second part of the Nation's Fund—viz., the provision for nurses who are in need—has been called the Tribute Fund, and I am glad to say that this part of the Fund also has progressed satisfactorily. It is especially in connection with this Tribute Fund that those ladies who were kind enough to help us in raising it have been subjected to most virulent and—as I think—disgraceful abuse. They have been told that they are insulting the nursing profession, and that the nurses do not want charity. I wish those who use this language could have seen, as I have, some of the cases that have been brought before the Committee dealing with the Tribute Fund and have received assistance from the Fund. I have a list of them here, of which I will give you two or three typical cases. I do not think I can do better than take the first three on the list. There are many other similar cases, and I shall be glad to show the list to anybody who wishes to see it.

A Few Typical Cases Assisted by the Tribute Fund Committee of the Nation's Fund for Nurses.

1. Staff nurse, Q.V.J.I.N. 1917-18, Q.A.I.M.N.S.R. 1915-17. Invalided from suffering from heart weakness and nervous debility. Discharged as permanently unfit, with gratuity of £60. No means, and mother partly dependent upon her. Tribute Fund Committee, Nation's Fund for Nurses, makes grant of 30s. per week.

2. Theatre sister and matron. Debility following malaria, para-typhoid, and dysentery contracted on service. Tribute Fund Committee, Nation's Fund for Nurses, makes grant of 30s. per week.

3. Staff nurse, T.F.N.S. Crippled with sclerosis, discharged November 1915 with gratuity of £110, now exhausted. Sole income 5s. per week from insurance. Tribute Fund Committee makes a grant of £1 per week, and is arranging for her to go to a permanent home.

You must remember that before we started this Nation's Fund there was practically no fund in existence that dealt with cases such as these, or at all events there were only very small funds, which were quite inadequate for the purpose. It was certain that after a war such as the one we have just been through, and after the very strenuous years of work on the part of the nurses, that there would be many cases of illness and breakdown that would require help, and this has actually proved to be the case. We all know how generous nurses are to one another, but after all the pay of nurses is still very small, and a nurse has all her work cut out during the years when she is strong and active to make provision for her own old age and possibly illness. She cannot spare much for others, and it is for this reason that we felt that in asking the nation to make provision for nurses in need of help we were only calling upon the people of this country to pay some part of the debt of gratitude that they owe to the nurses for their work not only in time of war, but also in time of peace.

State Registration.—You will have seen a great deal of comment in the papers about the two State Registration Bills which are at present before Parliament. The position is as follows: The Central Committee for State Registration were fortunate in the ballot and secured a day for the second reading of their Bill. As this Bill contains the principle of State Registration we decided to support the second reading, making it clear at the same time that the Bill was unsatisfactory from many points of view and would require amendment. We arranged for several amendments to be moved when the Bill was before Committee, but owing to the uncompromising attitude adopted by the promoters of the Bill, and to the fact that in such a short time we could not

**The College of Nursing**—(*continued*).

make our case fully known to the members of the Committee, the Bill was only slightly amended and was unsatisfactory in many ways when it left the Committee. We, therefore, decided that in order to present our case fully to Parliament it was desirable that our Bill should be introduced into the House of Lords, and this Lord Goschen kindly undertook to do. The Debate on the second reading was a very interesting one, and among those who took part in it were Lord Sandhurst, Lord Buckmaster, Lord Greville, and Lord Knutsford, who maintained his objection to State Registration, but said that he saw it was now inevitable but preferred to have a good Bill and not a bad one, giving this as a reason why he voted for the College Bill and not for that of the Central Committee.

The second reading of the Bill was passed by a large majority, and it has now reached the Committee stage. The net result, therefore, is that both Houses of Parliament have affirmed the principle of State Registration, and obviously the best solution would be for the Government itself to take the matter in hand. This is made easier by the fact that the Ministry of Health is now established, and, although I have no knowledge as to the intentions of the Government, we should be quite satisfied if they decided to take the matter in hand themselves. I do not wish to enter upon any of the controversial points connected with these two Bills, but I need only say that we urge, and shall continue to urge very strongly, that the College, with its 14,000 members, shall receive adequate representation on any provisional or permanent Committee that may be formed to deal with the Register, and that we are quite prepared to open our Register to inspection and to prove that we have on our books over 14,000 fully-trained certificated nurses. If the Central Committee for State Registration would only take this straightforward course in connection with their societies, and would show once and for all their membership and the qualifications for membership, much of the dissension in the nursing world would disappear. I have already stated that according to the best of my information the number of nurses represented by the Central Committee for State Registration and by the societies connected with it do not amount in all to half the number of nurses on the College Register, and I see no reason to alter my opinion. If I am doing an injustice to the Committee for State Registration they can very easily put the matter right by allowing any unbiased and competent person to examine their Registers and to say whether it is I or they who are speaking the truth.

At all events, the principle of State Registration has now been adopted by both Houses of Parliament, and the goal is within sight.

Before concluding, I must just say a few words on the work of the College. One very satisfactory feature mentioned in the Report is the establishment and success of the Local Centres that have been started throughout the country. We have now no less than nineteen of these, and others are in course of formation. These Centres will be invaluable not only because of the manner in which they will act as a rallying-place for nurses in every district, but also because they will keep the headquarters of the College in touch with nurses in every part of the country.

It is also mentioned in the Report that scholarships have already been given by the College, and it is contemplated to give more of these. This is only the beginning of the educational work which the College hopes to undertake.

We have also not been unmindful of the material welfare of the nurses. There was no question that in many cases the salaries were inadequate, their hours of work too long, and their conditions of service too onerous. Obviously, the first thing that we had to do was to investigate the matter and ascertain the true facts of the case. For this purpose we appointed a Salaries Committee: with the report of that Committee you are no doubt familiar. This Salaries Committee was composed entirely of people interested in the nursing profession, and who were not on the Council of the College of Nursing. Now that we have got the recommendations of the Committee, a small Sub-Committee of the College is carefully examining them, with a view to putting forward definite recommendations based on the report of the Salaries Committee. In this manner we hope to establish a general standard of wages, hours of work, and conditions of service to which, we trust, the training-schools throughout the country will endeavour to conform. I should like, in your name and in the name of the Council of the College of Nursing, to convey our very grateful thanks to Mrs. Field and the other ladies and gentlemen who gave so much time and trouble to the work of the Salaries Committee. You can judge from the Report itself how arduous that work was and how efficiently it was carried out. We also owe a great debt of gratitude to Miss Grant, the Secretary of that Committee, who performed her duties in the most capable and efficient manner.

There are other matters mentioned in the Annual Report, but I do not think it is necessary for me to occupy your time any longer. The year under review has been one of great interest to the nursing profession, and there has been a united movement forward in the direction of amelioration of the nurse's lot. The College does not wish to claim all the credit for this improvement, but it has undoubtedly contributed in no small measure to this happy result. You who are members of the College probably do not realise how much work is done by the Council and by the various Committees, composed, as they all are, of very busy people. I am sure, however, that you would wish me to convey to them your grateful thanks for the work that they have done and are doing on your behalf.

On the other hand, we look to you to help us, or rather to help yourselves, by doing all in your power to strengthen your own immediate Centre of the College and to do all you can to further its work. One of the best ways in which you can encourage and help us is by obtaining more members, and I still look to the figure of 20,000 nurses—which I mentioned last year—as the goal to be obtained before we can say that we are satisfied. I told you last year that if you would get 20,000 nurses, I would undertake that we should have not less than £20,000 in the bank. I am glad to think that I have more than redeemed my promise, and that, although you have not reached the figure of 20,000, our balance at the bank shows that we have got over £40,000 in hand. If you will each try to get one member—or, indeed, half a member will do—we shall reach the figure of 20,000 members this year, and I will do my best to secure a corresponding increase in the money standing to our credit at the bank.

I end, as I began, on a note of satisfaction with the progress of the College during the past three years, and especially during this past twelve months—a satisfaction in which I hope and believe you are all prepared to share.

